# Neuropsychiatric Symptoms in Behavioral Variant Frontotemporal Dementia and Alzheimer's Disease: A 12-Month Follow-Up Study

**DOI:** 10.3389/fneur.2021.728108

**Published:** 2021-09-30

**Authors:** Thais Bento Lima Da Silva, Tiago Nascimento Ordonez, Allan Gustavo Bregola, Valéria Santoro Bahia, Mário Amore Cecchini, Henrique Cerqueira Guimarães, Leandro Boson Gambogi, Paulo Caramelli, Marcio Luiz Figueredo Balthazar, Benito Pereira Damasceno, Sonia Maria Dozzi Brucki, Leonardo Cruz de Souza, Ricardo Nitrini, Monica Sanches Yassuda

**Affiliations:** ^1^Department of Neurology, Faculty of Medicine, University of São Paulo, São Paulo, Brazil; ^2^Gerontology, School of Arts, Sciences, and Humanities, University of São Paulo, São Paulo, Brazil; ^3^Graduate Program in Applied Statistics, University Center of United Metropolitan Colleges, São Paulo, Brazil; ^4^School of Health Sciences, Faculty of Medicine and Health Sciences, University of East Anglia, Norwich, United Kingdom; ^5^Human Cognitive Neuroscience, Psychology, University of Edinburgh, Edinburgh, United Kingdom; ^6^Neurology Division, University Hospital, Federal University of Minas Gerais, Belo Horizonte, Brazil; ^7^Neuroimaging Laboratory, School of Medical Sciences, University of Campinas (UNICAMP), Campinas, Brazil; ^8^Neurology Department, University of Campinas, Campinas, Brazil

**Keywords:** neuropsychiatric symptoms, behavioral dementia frontotemporal (bvFTD), Alzheimer's disease (AD), elderly, aging

## Abstract

**Introduction:** Neuropsychiatric symptoms in patients with frontotemporal dementia (FTD) are highly prevalent and may complicate clinical managements.

**Objective:** To test whether the Neuropsychiatry Inventory (NPI) could detect change in neuropsychiatric symptoms and caregiver's distress in patients diagnosed with behavioral variant frontotemporal dementia (bvFTD) and Alzheimer's disease (AD) from baseline to a 12-month follow-up and to investigate possible predictors of change in NPI scores.

**Methods:** The sample consisted of 31 patients diagnosed with bvFTD and 28 patients with AD and their caregivers. The Mini-Mental State Examination (MMSE), Addenbrooke's Cognitive Examination Revised (ACE-R), the INECO Frontal Screening (IFS), the Frontal Assessment Battery (FAB), the Executive Interview (EXIT-25) and the NPI were applied. Descriptive statistics, Mann-Whitney U test, Wilcoxon test, Chi square (χ^2^) test and Linear Regression Analysis were used.

**Results:** NPI total and caregiver distress scores were statistically higher among bvFTD patients at both assessment points. MMSE, ACE-R scores significantly declined and NPI Total and Distress scores significantly increased in both groups. In the bvFTD group, age was the only independent predictor variable for the NPI total score at follow up. In the AD group, ACE-R and EXIT-25, conjunctively, were associated with the NPI total score at follow up.

**Conclusions:** In 12 months, cognition declined and neuropsychiatric symptoms increased in bvFTD and AD groups. In the AD group only, cognitive impairment was a significant predictor of change in neuropsychiatric symptoms.

## Introduction

Behavioral variant frontotemporal dementia (bvFTD) is a neurodegenerative syndrome which is usually diagnosed in midlife (mean age at onset around 58 years). Prevalence peaks in the early sixties, at about 13 cases per 100,000 individuals. Among the frontotemporal dementias, bvFTD is the most common one as it represents 50% of the cases (1).

The diagnosis of bvFTD is a challenging one, mainly in the initial stage of the disease, when its clinical expression is limited to personality and behavioral changes (2). Close inspection of behavioral changes could support accurate differential diagnosis from psychiatric diseases and other dementias (3).

In bvFTD, identifying neuropsychiatric symptoms and following them up over time is relevant for treatment and disease management, as they may relate to the progressive decline in social and emotional functions. The frequency and intensity of such symptoms may alsohelp to distinguish bvFTD from other disorders. For instance, during bvFTD course, apathy can be the most frequent and intense symptom (2, 4).

In a previous study from our group (5), the most frequently reported symptoms among bvFTD patients were apathy (present in 85% of this patient group), irritability (65%), disinhibition (60%) and agitation/aggression (55%). Among patients with AD, depression (67%) and anxiety (63%) were most frequently reported. Those findings were in line with those from Riedijk et al. (6) and de Vugt et al. (7).

In a comparison between patients with bvFTD and Alzheimer's disease (AD), Kumfor et al. (8) reported that 60% of AD patients and 84% of bvFTD patients had apathy, and it was more severe and frequent in bvFTD patients. Besides, bvFTD patients presented higher affective and cognitive apathy, while AD patients presented only higher cognitive apathy. Findings on affective apathy were related to changes in the ventral prefrontal cortex areas, behavioral apathy was related to the basal ganglia and cognitive apathy was related to changes in the dorsomedial prefrontal cortex. In addition, the authors pointed out that care burden is an expected outcome of affective and behavioral apathy in bvFTD patients (8).

Clinical studies with follow-up data regarding neuropsychiatric symptoms in bvFTD and possible predictors of change are lacking (9). Therefore, we investigated whether there was significant change in neuropsychiatric symptoms and caregivers' distress in patients with bvFTD and AD, from baseline to the 12-month follow-up. We also investigated if there were associations between sociodemographic variables, cognitive performance and neuropsychiatric symptoms at both assessment points. This study is particularly important to describe clinical symptoms along the disease course in bvFTD and AD aiming to support treatment and disease management.

## Methods

### Materials

#### Demographic Information

Questions about age, sex, and years of education were answered by the caregivers.

#### Cognitive Assessment

University-based neurology outpatient services databases were queried, and patients and their family caregivers were recruited for the study. Specialized dementia centers across three Brazilian universities were involved: the Cognitive and Behavioral Neurology Group (GNCC-SP) and the Program for the Elderly (PROTER) at the University of São Paulo; the Cognitive and Behavioral Neurology Group (GNCC-MG) at the Federal University of Minas Gerais and the Department of Neurology at the State University of Campinas (UNICAMP).

#### Participants

A total of 59 individuals, comprising 28 diagnosed with AD and 31 with bvFTD, were included in the study. Patients with bvFTD and with AD were matched for disease severity on the Clinical Dementia Rating scale–frontotemporal lobar degeneration [CDR-FTLD, (10, 11)], with scores from 0 to 3.

The diagnosis of bvFTD and AD was performed by neurologists, geriatricians and psychiatrists, based on clinical, neurological history, neuropsychological assessments and screening for reversible causes of dementia along with laboratory and neuroimaging exams: functional Magnetic Resonance Imaging (fMRI) and Fluorodeoxyglucose PET (FDG-PET patterns). Dementia was diagnosed based on the criteria from the Diagnostic and Statistical Manual 5th Edition [DSM-V, (12)]. International diagnostic criteria were employed for diagnosing probable bvFTD (13). The National Institute on Aging - Alzheimer's Association (NIA/AA) criteria were used for AD diagnosis McKhann et al., (14).

Inclusion criteria for patients were age ≥40 years, education > 2 years and the presence of an informant who was involved in the daily routine of the patient (formal or informal carer; usually spending more than 8 h/day with the patient). Individuals presenting with visual, auditory or motor deficits preventing them from understanding instructions or performing cognitive tasks, individuals with other uncontrolled clinical diseases (such as hypertension and diabetes), serious and debilitating psychiatric disorders such as major depression, schizophrenia, bipolar disorder, clinical evidence or neuroimaging exam findings suggestive of vascular problems, dementias or etiologies other than bvFTD or AD, were excluded.

General cognition was assessed with the MMSE (0–30 points) [Folstein, (15)], (16) and the Addenbrooke's Cognitive Examination-Revised (ACE-R) [Mioshi et al., (17)], (18) (0–100 points).

Executive functions were assessed with the INECO Frontal Screening (IFS) (0–30 points), the Frontal Assessment Battery (FAB) (0–18 points) and the Executive Interview (EXIT-25) (0–50 points). The IFS items assess: response inhibition and set shifting [motor programming, conflicting instructions, go-no go test, verbal inhibitory control (Modified Hayling test)], abstraction (proverb interpretation) and, working memory (backward digit span, verbal working memory and spatial working memory). The IFS generates a separate score for working memory which varies from 0 to 9 (19, 20). The FAB is comprised of six subtests which assess conceptualization, mental flexibility, motor programming, sensitivity to interference, inhibitory control, and environmental mastery (21, 22). The EXIT-25 assesses verbal fluency, design fluency, anomalous sentence repetition, sensitivity to interference, among others (23, 24).

#### Neuropsychiatric Symptoms

The NPI assesses neuropsychiatric symptoms commonly found in dementia. It evaluates 12 domains (delusion, hallucinations, dysphoria, anxiety, agitation/aggression, euphoria, disinhibition, irritability/emotional lability, apathy, aberrant motor activity, night-time behavioral disturbances and appetite and eating abnormalities); thus yielding a composite symptom domain score (total score) (frequency × severity) ranging from 0 (absence of behavioral symptoms) to 144 points (maximum severity of behavioral symptoms) (25). The scale for assessing caregiver distress has scores ranging from 0 to 5 points (0 = no distress; 1 = minimal distress; 2 = mild distress; 3 = moderate distress; 4 = severe distress; and 5 = extreme distress) and the total distress score (NPI Distress) is calculated as the sum of the scores for each symptom.

### Statistical Analyses

Initially, all quantitative variables (continuous and discrete) were analyzed using the Kolmogorov-Smirnov test to assess whether or not they followed a normal distribution. The absence of normal distribution was observed in most quantitative variables, so non-parametric tests were used: Chi-square (x^2^) test, Mann-Whitney U test and Wilcoxon test.

The Mann-Whitney U test was used, at different times, to compare bvFTD vs. AD groups. To analyze the differences between baseline and the 12-month follow up within the same clinical group, the Wilcoxon test was used. To analyze the influence of sociodemographic and cognitive variables on the NPI scores, linear regression analysis was used, with a multivariate model, and stepwise forward criteria for the selection of independent variables (age, gender, years of education, MMSE, ACER, EXIT-25, IFS, FAB), from the simplest to the most complex model (26).

The computer program Statistica 7.0 was used. The level of significance adopted for the statistical tests was 5%, that is, *p*-value < 0.05.

### Procedures and Ethical Aspects

This study was approved by the Ethics Committee of the Hospital das Clínicas, protocol number 311.601. The study was conducted in compliance with international ethical standards, according to the Declaration of Helsinki.

## Results

The demographic and clinical characteristics of the patients are presented in Table 1. At baseline, 29 men (49.15%) and 30 women (50.85%) were included in the study. The mean age was 70.29 ± 9.85 years (range 50–87 years).

**Table 1:** Demographic and clinical characteristics of patients diagnosed with Alzheimer's disease (AD) and behavioral variant frontotemporal dementia (bvFTD).

**Characteristics**	**bvFTD (*****n*** **= 31)**		**AD (*****n*** **= 28)**		
	**Mean**	**SD**	**Mean**	**SD**	***p-value***
Women *n* (%)	13 (41.90%)		17 (60.71%)		0.195^a^
Age (range: 50–87)	66.94	9.26	74.15	9.22	0.004^b^
Schooling (0–21 Years)	11.74	4.57	9.43	4.49	0.055^b^
**Clinical characteristics**					
MMSE (Baseline)	23.61	4.96	23.35	3.54	0.397^b^
MMSE (1 Year)	22.50	4.69	22.87	3.91	0.775^b^
	0.005 ^c^		0.043^c^		
ACER (Baseline)	71.71	16.36	67.57	11.77	0.173^b^
ACER (1 Year)	69.32	15.54	66.61	11.51	0.563^b^
	0.001^c^		0.028^c^		
EXIT-25 (Baseline)	14.96	9.36	12.43	7.82	0.353^b^
EXIT-25 (1 Year)	16.74	10.28	12.90	9.04	0.256^b^
	0.028^c^		0.753^c^		
IFS (Baseline)	15.42	6.35	16.41	5.06	0.973^b^
IFS (1 Year)	15.02	6.34	16.06	5.13	0.942^b^
	0.423^c^		0.108^c^		
FAB (Baseline)	12.42	4.07	13.75	2.69	0.607^b^
FAB (1 Year)	12.35	4.02	13.60	2.74	0.386^b^
	1.00^c^		1.00^c^		
NPI Total (Baseline)	45.58	23.85	25.64	16.92	0.001^b^
NPI Total (1 Year)	47.90	22.88	28.36	19.46	0.001^b^
	0.008^c^		0.043^c^		
NPI Distress (Baseline)	19.16	10.19	12.29	8.20	0.007^b^
NPI Distress (1 Year)	20.13	9.76	13.11	8.63	0.006^b^
	0.005^c^		0.028^c^		

a *Chi-square Test*.

b *Mann-Whitney U Test*.

c *Wilcoxon Matched Pairs Test*.

*AD, Alzheimer's disease; bvFTD, behavioral variant frontotemporal dementia; MMSE, Mini Mental State Examination; ACE-R, Addenbrooke's Cognitive Examination-Revised; EXIT-25, Executive Interview with 25 items; IFS, INECO Frontal Screening; FAB, Frontal Assessment Battery*.

Patients with AD were significantly older than the patients with bvFTD. MMSE and ACE-R scores decreased significantly from baseline to follow-up in both clinical groups. EXIT-25 scores significantly declined for the bvFTD group only, indicating that the executive dysfunction may have increased over time in this group. IFS and FAB scores remained unchanged for both groups (Table 1). NPI Total and Caregiver Distress scores were significantly higher for the bvFTD group at both assessment times. For both clinical groups, NPI Total and Distress scores significantly increased from baseline to follow up.

**Figure 1:**
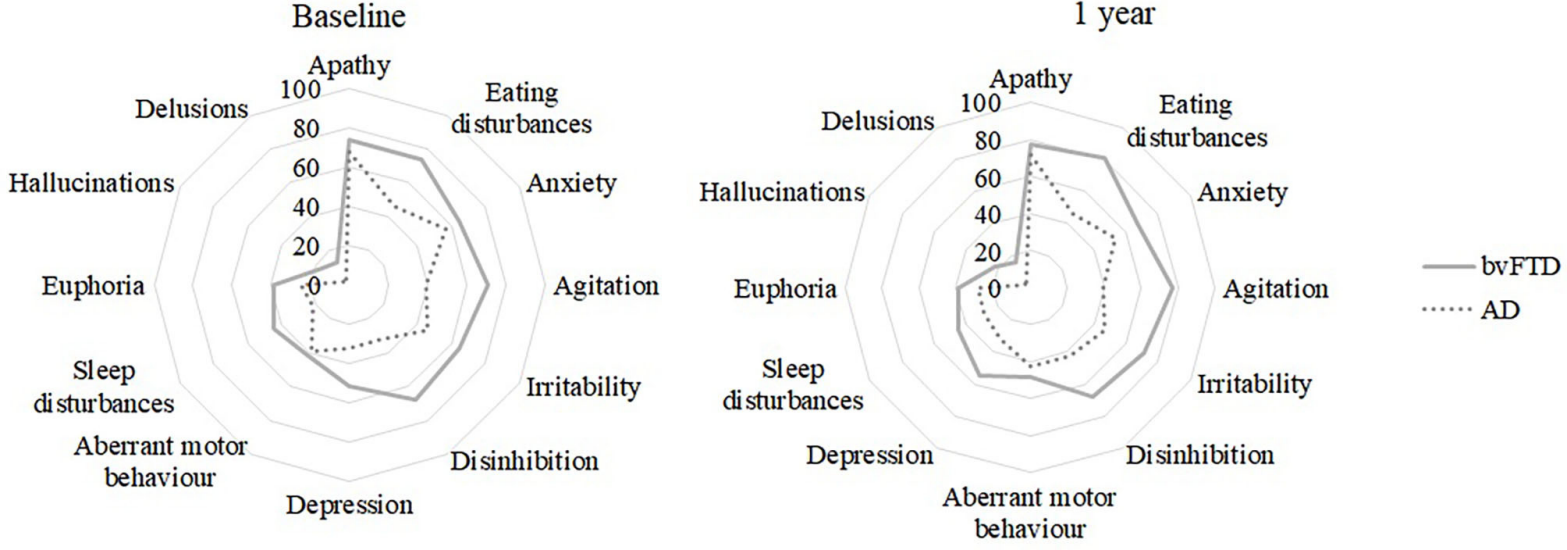
Frequency (%) of patients with neuropsychiatric symptoms (baseline and 1-year follow-up) for each clinical group.

For a better graphical display of the NPI results, a radar chart was used (Figure 1). In this type of chart, the value axes start from a common center. For this study, the vertical main axis represents the 12 dimensions of the NPI. A line connects the score obtained in each assessment, forming a polygon. The scores obtained at baseline and at follow-up by different groups can be easily compared by looking at the area of the 12-sided polygon. The larger the area of the polygon, the higher the reported symptoms. The shape of the polygon is also relevant, since asymmetries indicate that there are differences in the investigated domains.

When the clinical groups were compared at baseline, significant differences emerged, as higher scores can be seen for the bvFTD group for: agitation, eating disturbances and disinhibition (Figure 1; Table 2), the latter two were also observed in the NPI Distress subdomains (Table 3).

**Table 2:** Mean neuropsychiatric inventory scores reported by caregivers for each symptom.

**Characteristics**		**bvFTD (*****n*** **= 31)**		**AD (*****n*** **= 28)**		
		**Mean**	**SD**	**Mean**	**SD**	***p*-value**
Delusions	Baseline	0.84	2.45	0.11	0.57	0.192^a^
	1 year	0.97	2.51	0.14	0.76	0.108^a^
		1.000^b^		1.000^b^		
Hallucinations	Baseline	1.00	2.61	0.21	1.13	0.105^a^
	1 year	1.26	2.74	0.29	1.51	0.032^a^
		1.000^b^		1.000^b^		
Agitation	Baseline	6.03	4.61	3.21	4.55	0.031^a^
	1 year	6.61	4.43	3.32	4.72	0.009^a^
		1.000 ^b^		0.789^b^		
Depression	Baseline	2.90	3.62	2.18	3.57	0.245^a^
	1 year	3.29	3.87	2.00	3.33	0.119^a^
		0.248^b^		1.000^b^		
Anxiety	Baseline	4.32	4.17	4.00	4.32	0.693^a^
	1 year	4.58	4.07	3.68	4.27	0.356^a^
		1.000^b^		1.000^b^		
Euphoria	Baseline	2.81	3.99	1.21	2.50	0.148^a^
	1 year	2.58	3.82	1.54	2.89	0.322^a^
		1.000^b^		1.000^b^		
Apathy	Baseline	6.29	4.41	4.32	4.15	0.103^a^
	1 year	6.35	4.32	4.75	4.30	0.187^a^
		1.000^b^		1.000^b^		
Disinhibition	Baseline	5.13	4.54	2.18	3.75	0.005^a^
	1 year	5.16	4.56	3.04	4.10	0.044^a^
		1.000^b^		0.109^b^		
Irritability	Baseline	5.00	4.37	2.89	3.93	0.075^a^
	1 year	5.29	4.27	3.04	4.04	0.042^a^
		1.000 ^b^		0.789^b^		
Aberrant motor behavior	Baseline	2.94	4.11	1.50	2.56	0.333^a^
	1 year	3.26	4.07	2.04	3.28	0.311^a^
		1.000^b^		0.109^b^		
Sleep disturbances	Baseline	2.77	3.66	1.29	3.03	0.056^a^
	1 year	2.84	3.66	1.93	3.70	0.230 ^a^
		1.000^b^		1.000^b^		
Eating disturbances	Baseline	5.45	4.20	2.46	3.49	0.007^a^
	1 year	5.71	3.97	2.75	3.92	0.005^a^
		1.000^b^		1.000^b^		

a *Mann-Whitney U Test*.

b *Wilcoxon Matched Pairs Test*.

**Table 3:** Mean neuropsychiatric inventory distress reported by caregivers for each domain.

**Characteristics**		**bvFTD (*****n*** **= 31)**		**AD (*****n*** **= 28)**		
		**Mean**	**SD**	**Mean**	**SD**	***p*-value**
Delusions	Baseline	0.35	0.98	0.07	0.38	0.209^a^
	1 year	0.42	1.03	0.14	0.76	0.121^a^
		1.000^b^		1.000^b^		
Hallucinations	Baseline	0.45	1.21	0.11	0.57	0.111^a^
	1 year	0.55	1.29	0.18	0.94	0.034^a^
		1.000^b^		1.000^b^		
Agitation	Baseline	2.55	1.80	1.54	2.03	0.074^a^
	1 year	2.81	1.70	1.50	1.99	0.018^a^
		1.000^b^		1.000^b^		
Depression	Baseline	1.58	1.73	1.00	1.52	0.188^a^
	1 year	1.68	1.72	0.89	1.47	0.069^a^
		1.000^b^		1.000^b^		
Anxiety	Baseline	1.84	1.61	1.61	1.64	0.620^a^
	1 year	2.06	1.59	1.50	1.64	0.205^a^
		1.000^b^		1.000^b^		
Euphoria	Baseline	1.06	1.55	0.75	1.38	0.380^a^
	1 year	0.94	1.46	0.82	1.39	0.730^a^
		1.000^b^		1.000^b^		
Apathy	Baseline	2.52	1.81	2.29	1.80	0.700^a^
	1 year	2.55	1.77	2.43	1.77	0.881^a^
		1.000^b^		1.000^b^		
Disinhibition	Baseline	2.16	1.92	1.18	1.87	0.023^a^
	1 year	2.19	1.96	1.61	2.02	0.172^a^
		1.000^b^		0.109^b^		
Irritability	Baseline	1.97	1.68	1.46	1.79	0.317^a^
	1 year	2.03	1.64	1.50	1.86	0.248^a^
		1.000^b^		0.789^b^		
Aberrant motor behavior	Baseline	1.10	1.56	0.96	1.62	0.676^a^
	1 year	1.26	1.57	1.04	1.62	0.527^a^
		1.000^b^		1.000^b^		
Sleep disturbances	Baseline	1.29	1.70	0.54	1.20	0.062^a^
	1 year	1.32	1.76	0.82	1.49	0.248^a^
		1.000^b^		1.000^b^		
Eating disturbances	Baseline	2.23	1.65	0.75	1.29	0.001^a^
	1 year	2.29	1.60	0.82	1.42	0.001^a^
		1.000^b^		1.000^b^		

a *Mann-Whitney U Test*.

b *Wilcoxon Matched Pairs Test*.

The groups were significantly different at baseline and follow-up, with higher scores for the bvFTD group, in NPI agitation, disinhibition, and eating disturbances, see Figure 1 and Table 2. For NPI hallucination and irritability significant differences between the groups emerged only at follow up. For the NPI distress, hallucinations, agitation and eating disturbances scores were significantly higher for the bvFTD group at follow up (Table 3).

We did not find significant correlations between NPI data and cognitive variables. However, using the Linear Regression Analysis, as seen in Tables 4, 5, age was the only independent predictor variable for the NPI Total score in the bvFTD Group in the follow up. And in the AD group, ACE-R and EXIT-25 (follow-up) were associated with the NPI Total score in the follow-up.

**Table 4:** Linear regression analysis for NPI total score (baseline and 1-year follow-up) among bvFTD patients.

**Dependent variable**	**Independent variables**	**Beta**	**Std.Err**.	***p*-level**
NPI Total (baseline)^a^	Age	−0.330	0.197	0.107
NPI Total (1 Year)^b^	Age	−0.464	0.200	0.030
	Schooling	−0.209	0.200	0.306

a *R = 0.330, R^2^ = 0.108, Adjusted R^2^ = 0.070; F_(1, 23)_ = 2.810 p < 0.107 Std. Error of estimate: 23.089*.

b *R = 0.445, R^2^ = 0.199, Adjusted R^2^ = 0.127, F_(2, 22)_ = 2.7453 p < 0.086 Std. Error of estimate: 21.467. Dependent variables: Total NPI (baseline and follow-up). Independent variables: sex (1 = woman; 0 = man), age, education, MMSE, ACER, EXIT-25, IFS, FAB*.

**Table 5:** Linear regression analysis for NPI total score (baseline and 1-year follow-up) among AD patients.

**Dependent variable**	**Independent variables**	**Beta**	**Std. Err**.	***p*-level**
NPI Total (baseline)^a^	Schooling	−0.283	0.266	0.307
NPI Total (1 Year)^b^	ACE-R Total	−0.623	0.290	0.045
	EXIT-25	−0.658	0.289	0.035

a *R = 0.283, R^2^ = 0.080, Adjusted R^2^ = 0.009, F_(1, 13)_ = 1.131 p < 0.307 Std. Error of estimate: 17.119*.

b *R = 0.494, R^2^ = 0.244, Adjusted R^2^ = 0.159, F_(2, 18)_ = 0.904 p < 0.080 Std. Error of estimate: 17.947. Dependent variables: Total NPI (baseline and follow-up). Independent variables: sex (1 = woman; 0 = man), age, education, MMSE, ACER, EXIT-25, IFS, FAB*.

## Discussion

The aim of the present study was to test the hypothesis that there was significant change in neuropsychiatric symptoms, assessed by the NPI, in patients with bvFTD and AD, from baseline to the 12-month follow-up. We also investigated if there were changes in the NPI Caregiver Distress score and explored potential links between sociodemographic variables, cognitive performance and neuropsychiatric symptoms at baseline and follow-up. The groups were statistically similar in terms of sex, education, cognitive and functional assessment scores. The mean age was higher in the AD group. The clinical groups differed from the start in terms of NPI Total and Distress scores (bvFTD > AD).

In the present study, after 12 months, both groups presented with a reduction in MMSE and ACE-R scores and an increase in the NPI Total and Distress scores. An increase in executive dysfunction was also observed, according to the EXIT-25 scores, in the bvFTD group.

Neuropsychiatric symptoms (NPI Total and Distress scores) were statistically higher among bvFTD patients. Separately, agitation, disinhibition and eating disorders symptoms were higher in the bvFTD group, at baseline and follow-up assessments. Hallucination, agitation and irritability were higher in bvFTD at follow-up assessment in NPI Distress. These data confirm previous studies results (27, 28) as they indicate higher severity of NPI symptoms in bvFTD than in AD.

Not many studies have looked at differences between dementia subtypes in clinic-based samples using the NPI. In the first study that looked at differences in the NPI between AD and bvFTD, disinhibition, euphoria, apathy and aberrant motor behavior were found to be significantly higher in FTD (29). The same differences were noted in an Italian sample of patients with AD and FTD [Leroi et al., (30)]. Mendez et al. (31) had also observed higher scores for FTD patients in the verbal outbursts and inappropriate activity subscales of the BEHAVE-AD rating scale, while AD patients had higher scores on the affective disturbance and anxieties/phobias subscales.

In a recent study, with bvFTD, AD patients, and primary progressive aphasia (PPA) patients, Radakovic et al. (32) used the Dimensional Apathy Scale (DAS), which assesses: executive, emotional and initiation apathy. A total of 12 patients with PPA, 12 with bvFTD, and 28 with AD, and their caregivers (or relatives and close friends) answered the DAS and the apathy subtype awareness was obtained by the caregivers, to assess the discrepancy rate. There was higher emotional apathy and lower awareness for emotional apathy in bvFTD patients than in AD patients (32).

Liu et al. (28) suggested that neuropsychiatric symptoms are significant predictors of institutionalization (28). In bvFTD, patients' caregivers seem to experience higher levels of burden and suffering than AD patients' caregivers do. Neuropsychiatric symptoms seem to be associated with greater burden and suffering in bvFTD patients' caregivers, as observed in the present study and previous ones (2, 5).

There is limited information regarding the trajectory of neuropsychiatric symptoms over time in AD and bvFTD. Present results suggest there was significant worsening in NPI (Total and Distress) in both groups. For some NPI domains, group differences reached significance at follow up, with worse scores in the bvFTD group, which suggests changes in NPI scores were of higher magnitude in this group. These results, in a short follow up period, suggest that it is relevant to track changes in neuropsychiatric symptoms over time, to better caregivers regarding care challenges. Higher emotional overload may be present in bvFTD patients' caregivers, due to behavior and personality changes, as assessed with the NPI scale (5). Additionally, studies have reported the difficulty of caregivers of patients with bvFTD in managing day-to-day cognitive and behavioral impairments (33).

Finally, we highlight that neuropsychiatric symptoms in AD only were associated with cognitive scores in the regression analyses. This finding may perhaps be explained by the fact that cognitive impairment is a core symptom in AD since the early disease stages and, therefore, cognition may drive neuropsychiatric symptoms.

As to study limitations, we cite that the present study was based on relatively small samples, and this may have hindered the identification of group differences of small magnitude. As to its strengths, we indicate the inclusion of a follow up assessment.

Due to the epidemiological significance of bvFTD, further research studies on the clinical characterization of the disease course are needed. Research studies with larger samples, including different dementia subtypes, examining the links between cognitive performance, neuropsychiatric symptoms and caregiver burden are recommended.

## Data Availability Statement

The original contributions presented in the study are included in the article/supplementary material, further inquiries can be directed to the corresponding author/s.

## Ethics Statement

The studies involving human participants were reviewed and approved by the Ethics Committee for Analysis of Research Projects (CAPPesq) of the Medical Board of the Clinics Hospital and of the University of São Paulo School of Medicine, protocol number 311.601. The study was conducted in compliance with international ethical standards (Declaration of Helsinki). The patients/participants provided their written informed consent to participate in this study.

## Author Contributions

All authors listed have made a substantial, direct and intellectual contribution to the work, and approved it for publication.

## Funding

This project was supported by the São Paulo Research Foundation (FAPESP) Grant Number: 11/04804-1 and 16/07967-2. Conselho Nacional de Desenvolvimento Científico e Tecnológico (CNPq), Grant Number: 151684/2014-6 and by Coordenação de Aperfeiçoamento de Pessoal de Nível Superior (CAPES), Grant Number: 88881.131619/2016-01.

## Conflict of Interest

The authors declare that the research was conducted in the absence of any commercial or financial relationships that could be construed as a potential conflict of interest.

## Publisher's Note

All claims expressed in this article are solely those of the authors and do not necessarily represent those of their affiliated organizations, or those of the publisher, the editors and the reviewers. Any product that may be evaluated in this article, or claim that may be made by its manufacturer, is not guaranteed or endorsed by the publisher.
